# Arctiin attenuates high glucose‐induced human retinal capillary endothelial cell proliferation by regulating ROCK1/PTEN/PI3K/Akt/VEGF pathway in vitro

**DOI:** 10.1111/jcmm.15232

**Published:** 2020-04-16

**Authors:** Min Zhou, Guobing Li, Liancai Zhu, Huyue Zhou, Laichun Lu

**Affiliations:** ^1^ Key Laboratory of Biorheological Science and Technology Ministry of Education College of Bioengineering Chongqing University Chongqing China; ^2^ Department of Pharmacy Xinqiao Hospital Army Medical University Chongqing China

**Keywords:** arctiin, diabetic retinopathy, HRCECs, Rho associated coiled‐coil containing protein kinase 1, VEGF

## Abstract

Diabetic retinopathy (DR) is one of the most prominent microvascular complications of diabetes, which remains the leading cause of legal blindness in the world. Arctiin, a bioactive compound from *Arctium lappa* L., has been reported to have antidiabetic activity. In this study, we investigated the effect of arctiin on a human retinal capillary endothelial cell (HRCEC) line and how arctiin inhibits cell proliferation in high glucose (HG)‐induced HRCECs. Results showed that arctiin decreased HG‐induced HRCECs proliferation in a dose‐dependent manner by inducing cell cycle arrest at the G0/G1 phase. Tube formation assay and immunofluorescence staining indicated that arctiin abrogated tube formation induced by HG‐induced HRCECs in a dose‐dependent manner via down‐regulation of VEGF expression. Mechanistic study indicated that perturbation of the ROCK1/PTEN/PI3K/Akt signalling pathway plays a vital role in the arctiin‐mediated anti‐proliferative effect. Furthermore, pre‐incubation of HRCECs with Y‐27632 attenuated arctiin‐induced cell cycle arrest, cell proliferation and tube formation inhibition. Y‐27632 also reversed the activation of PTEN, the inactivation/dephosphorylation of PI3K/Akt and down‐regulation of VEGF. Taken together, the results demonstrated that arctiin inhibits the proliferation of HG‐induced HRCECs through the activation of ROCK1 and PTEN and inactivation of PI3K and Akt, resulting in down‐regulation of VEGF, which inhibits endothelial cell proliferation.

## 1 INTRODUCTION

Diabetic retinopathy (DR) is a prominent microvascular complication of diabetes mellitus, which remains the leading cause of preventable blindness.^1^ Diabetic retinopathy is characterized by a breakdown of the blood‐retinal barrier, a loss of pericytes from retinal capillaries, the appearance of acellular capillaries, and retinal neovascularization, which greatly increases the probability of vision loss.^2^, ^3^, ^4^ Currently, intravitreal injections of glucocorticoids and anti‐VEGF drugs are popular clinical therapeutics but still have some limitations.^5^ A considerable proportion of patients do not respond to these methods.^6^ Therefore, seeking novel therapy and drugs for DR is particularly important to control the development of the disease and to reduce the rate of blindness. Clinically, early treatment of DR is carried out by controlling blood glucose and blood pressure. However, the "high blood glucose memory" effect prevents tight glucose control from completely preventing the occurrence and development of DR. Therefore, the development of novel and valid therapy and drugs for the early pathogenesis of DR is a popular topic in current DR research.

Rho‐associated coiled‐coil kinase 1 (ROCK1, 160 kDa) belongs to a family of serine/threonine kinases that are activated via interaction with Rho GTPases or caspase‐3 by cleavage the C‐terminal autoinhibitory domain away (30 kDa) from the kinase active site.^7^ ROCK is involved in a wide range of fundamental cellular functions, such as cellular adherence, migration, proliferation and apoptosis.^8^ Recent studies demonstrated that ROCK modulates hyperglycaemia‐induced microvascular endothelial dysfunction,^9^, ^10^ suggesting a potential target for the treatment of DR. Phosphatase and tensin homologue deleted on chromosome ten (PTEN) is an important ROCK substrate,^11^ and the phosphorylation of PTEN by ROCK stimulates its phosphatase activity. PTEN is an antagonist regulator of the phosphatidylinositol (PI)3‐kinase/Akt pathway, which is known to play a major role in a diverse range of biological processes, including angiogenesis, cell survival and cell death.^12^, ^13^ Studies have shown that the PI3K/Akt pathway promotes angiogenesis by activating vascular endothelial growth factor (VEGF) expression.^14^

Arctiin, an herb widely used in traditional Chinese medicine for the treatment of the common cold or flu, is a lignin bioactive compound isolated from the dry seeds of *Arctium lappa* L.^15^ This lignin compound has been known to exhibit anti‐inflammatory, anti‐proliferative and antimicrobial activity.^16^, ^17^, ^18^, ^19^ It has also been shown to inhibit 2‐amino‐3, 8‐dimethylimidazo [4,5‐f]quinoxaline (MeIQx)‐induced hepatocarcinogenesis and 2‐amino‐1‐methyl‐6‐phenylimidazo[4,5‐b]pyridine (PhIP)‐induced mammary, colon and pancreatic carcinogenesis.^20^ Additionally, some studies have reported that arctiin can protect the glomerular filtration barrier, can effectively relieve microcirculatory disturbances and show a good therapeutic effect on diabetic nephropathy (DN).^21^ Our previous studies indicated that arctiin decreases the severity of diabetic complications and might be used as an effective inhibitor of diabetic retinopathy.^22^ However, the precise mechanism of the antidiabetic activity of arctiin has not been fully explored.

In the present study, the HRCECs were used to investigate the inhibitory effects of arctiin on cell proliferation. Arctiin‐inhibited HG‐induced cell proliferation in HRCECs and induced cell cycle arrest at the G0/G1 phase. Additionally, arctiin abrogated HG‐induced tube formation of HRCECs. Moreover, mechanistic study indicated that arctiin‐inhibited HG‐induced HRCEC proliferation through the activation of ROCK1 and PTEN and the inactivation of PI3K and Akt, resulting in down‐regulation of VEGF. Furthermore, pre‐incubation of cells with Y‐27632, a specific ROCK1 inhibitor, dramatically attenuated the anti‐proliferative activity of arctiin. In addition, Y‐27632 attenuated arctiin‐induced VEGF down‐regulation and G0/G1 phase cell cycle arrest. Our study provides novel insight into anti‐proliferative effect of arctiin and suggests that arctiin may be a promising agent for the treatment of DR.

## 2 MATERIALS AND METHODS

### 2.1 Chemicals and antibodies

Arctiin (Purity: 99% by HPLC, Appearance: white crystal power) was manufactured by Chongqing Kerui Pharmaceutical Co., Ltd. (Chongqing, China). Y‐27632 was purchased from Santa Cruz Biotechnology (Santa Cruz, CA). 3‐(4,5‐methylthiazol‐2‐yl)‐2,5‐diphenyltetrazolium bromide (MTT) was obtained from Amresco (Solon, Ohio, USA). The following antibodies were used: anti‐PTEN (A2B1, sc‐7974, Santa Cruz, 1:500), anti‐CDK2 (55, sc‐136191, Santa Cruz, 1:500), anti‐CDK4 (3F121, sc‐70831, Santa Cruz, 1:500), anti‐Cyclin D1 (H‐295, sc‐753, Santa Cruz, 1:500), anti‐PI3K (p110α, #4255, Cell Signaling Technology, 1:1000), anti‐Phospho‐PI3K (Tyr458,#4228, Cell Signaling Technology, 1:1000), anti‐Phospho‐Akt (Thr308, #9275, Cell Signaling Technology, 1:500), anti‐Akt (#9272, Cell Signaling Technology, 1:1000), anti‐ROCK1 (EP786Y, ab45171, abcam, 1:1000), anti‐β‐actin (AC‐15,#1978,Sigma,1:50 000), anti‐Ki67 (ab15580, Abcam, 1:1000), Human VEGF PicoKine ELISA Kit (Boster, China) and p‐VEGFR2 (Tyr1175, #2478, Cell Signaling Technology, 1:1000); VEGFR2 (D5B1, #9698S, Cell Signaling Technology, 1:1000).

### 2.2 Cell culture and identification

Human retinal capillary endothelial cells (HRCECs) were purchased from Guangzhou Jennio Biotech Co., Ltd. (Guangzhou, China). The cells were cultured in Dulbecco's modified Eagle's medium (DMEM, Gibco, USA) with 10% foetal bovine serum (FBS, Hyclone, USA) and 1% penicillin/streptomycin (HyClone, USA). Cells were cultured in an incubator at 37°C in a humidified atmosphere of 5% CO_2_ and 95% air. For all cell experiments, only primary cells at passages 2‐10 were used.

### 2.3 Arctiin treatments

The HRCECs were cultured in DMEM of 1.0 g/L glucose as the normal group (NG) and 4.5 g/L glucose (HG) as the model group. Subsequently, the cells of the arctiin treatment groups incubated in DMEM with 4.5 g/L glucose were simultaneously stimulated with different concentrations of arctiin (0.05, 0.5 or 2.0 mg/mL) for 48 hours. The treated cells were used in cell viability studies, as well as in the clonogenicity assay, the tube formation assay, immunofluorescence staining, flow cytometry and Western blot analysis.

### 2.4 MTT assay

The MTT assay was performed for assessing cell viability after treatment. HRCECs were seeded at a density of 1 × 10^4^ cells per well in 96‐well plates and grown to approximately 70% confluence prior to drug treatment. The cells were cultured in DMEM with 1.0 g/L glucose as the control group and with 4.5 g/L glucose as the model group. The cells of the arctiin‐treated groups were incubated in DMEM with 4.5g/L glucose and different concentrations of arctiin (0.05, 0.5 or 2.0 mg/mL). Following incubation for 48 hours, 20 μL of MTT (5 mg/mL) was added to each well for 4 hours at 37°C. Then, 150 μL of DMSO was added to dissolve the formazan crystals, and the absorbance of each plate was measured at 490 nm using a Spectra Max Plus microplate reader (Molecular Devices).

### 2.5 Clonogenicity assay

Cells were seeded at a density of 100 cells per dish in 5 cm dishes and cultured in DMEM with 10% FBS and 1% penicillin/streptomycin. After treatment with different concentrations of arctiin, the cells were cultured for 2‐3 weeks, and all media were changed every five days. Then, the clones were fixed with 4% paraformaldehyde for 15 minutes and were stained with crystal violet for 10 minutes. The images were acquired with a digital camera.

### 2.6 Immunofluorescence staining

The treated cells were seeded onto slides and cultured in 24‐well plates at a density of 5 × 10^5^ cells per well. After 48 hours, the cells were fixed with 1 mL of immunol staining fix solution (Beyotime, P0098) for 15 minutes, permeabilized with 0.1% Triton X‐100 for 5 minutes, blocked with 5% FBS for 30 minutes and incubated with Ki‐67 primary antibody and then with Alexa Fluor 647 goat anti‐Rabbit secondary antibody (Invitrogen, A21245,1:300). The nuclei were stained with DAPI, and the cells were visualized by laser‐scanning confocal microscope (LSM780NLO, Zeiss, Germany). The quantification of Ki67‐positive cells was calculated by Zeiss LSCM Image Examiner software, and three images were examined in our experiments.

### 2.7 Tube formation assay

Briefly, the 96‐well plates were precoated with 50 µL Matrigel (BioFroxx, 1567ML005) per well and were then placed at 37°C for 30 minutes to solidify. HRCECs (5 × 10^4^ cells/well) in different treatment groups were seeded on the solidified Matrigel. The plates were maintained at 37°C, under a humidified atmosphere of 5% CO_2_ for 24 hours. The pictures were captured with a microscope. The quantification of total tube length was calculated by ImageJ software using the ‘Angiogenesis analyze’ tool.

### 2.8 Cell cycle analysis

The cell cycle was analysed using a flow cytometry‐based method. The treated HRCECs were digested, collected and subsequently washed with ice‐cold PBS. The obtained cells were fixed in 75% cold ethanol for 8 hours at 4°C. The fixed cells were washed twice with PBS and incubated with RNase (50 μg/mL) and propidium iodide (PI) (50 μg/mL, Thermo Fisher) for 30 minutes. The results were measured by flow cytometry (FACScan, BD Biosciences), and the percentages of cells in G0/G1, S and G2/M phases were analysed by FlowJo software.

### 2.9 Western blot

After treatment with arctiin, the HRCECs were lysed with RIPA lysis buffer (Beyotime, Shanghai, China), and the protein concentrations were measured using an enhanced BCA protein assay kit (Beyotime, Shanghai, China). The proteins were separated by SDS‐PAGE gels and were then transferred onto PVDF membranes (Millipore, Massachusetts, USA). The membranes were blocked with 5% non‐fat milk at 37°C for 2 hours and were then incubated overnight with the corresponding primary antibodies at 4°C. After rinsing 3 times with 1 × TBST, the membranes were then incubated with a horseradish peroxidase‐bound secondary antibody for 2 hours. Enhanced chemiluminescence reagents were added for the visualization of the protein membranes.

### 2.10 ELISA assay

The protein concentrations of VEGFA in HRCECs supernatant samples were determined with a commercial ELISA kit (Boster, China) according to the manufacturer's instructions. Absorbance was detected at 450 nm in a microplate reader.

### 2.11 Statistics

All quantitative data were reported at least three separate experiments performed in triplicate, unless otherwise noted. Statistical significance was evaluated by Student's *t* test. For comparisons of more than two groups, one‐way ANOVA followed by post hoc t ‐tests was performed, and the levels of significance were set as *P* < .05.

## 3 RESULTS

### 3.1 Arctiin inhibits HG‐induced cell proliferation and tube formation in HRCECs

**FIGURE 1:**
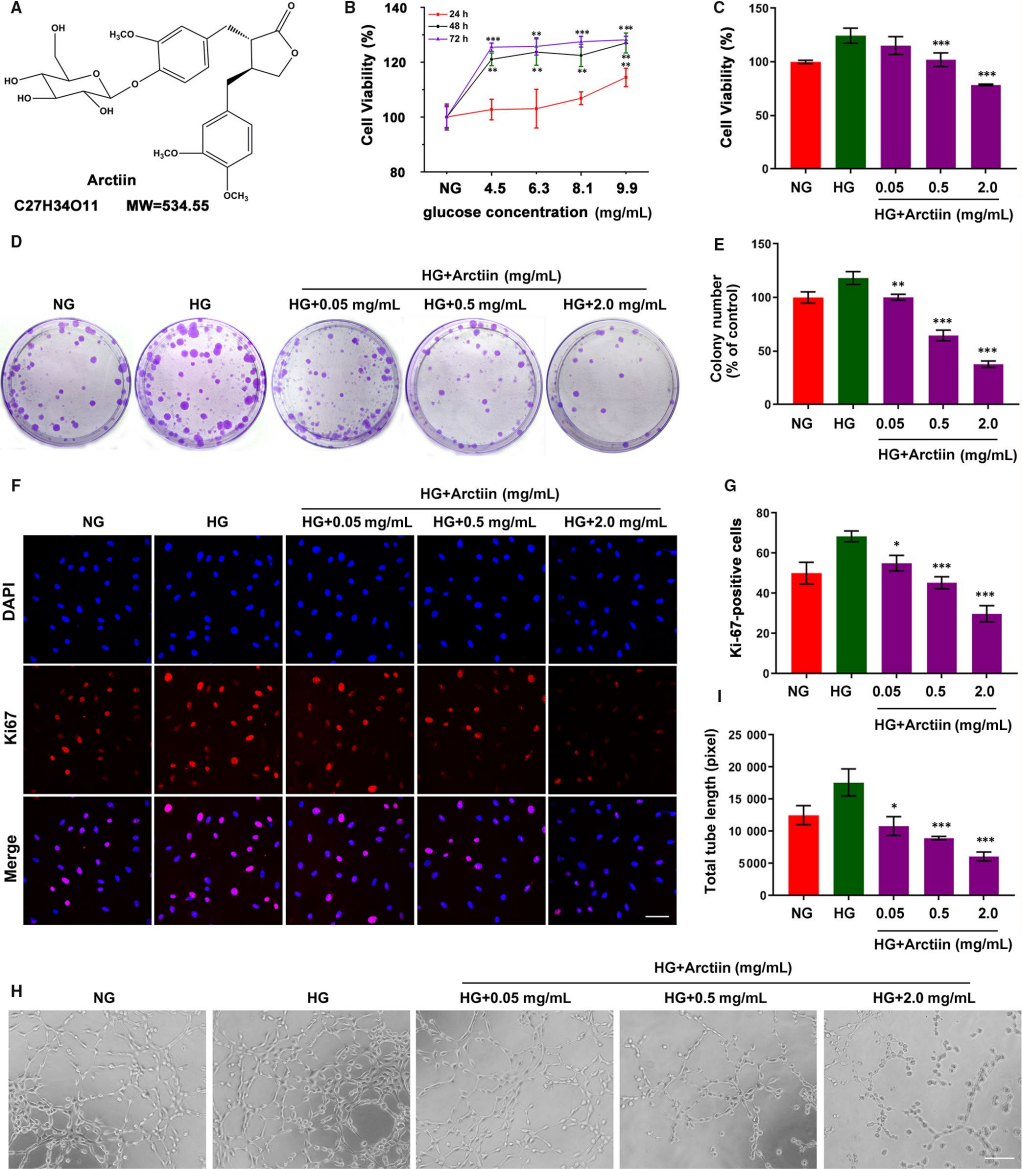
Arctiin inhibits HG‐induced cell proliferation and tube formation in HRCECs. (A) Chemical structure and molecular weight of arctiin. (B) Effect of different high glucose (HG) concentrations (4.5, 6.3, 8.1, 9.9 mg/mL) on cell viability at different time‐points in HRCECs. (C) HRCECs were treated with HG (4.5 mg/mL) without or with different concentrations of arctiin (0.05, 0.5 or 2.0 mg/mL) for 48 h, and cell viability was detected by MTT assay. (D‐E) The anti‐proliferative activity of arctiin on HG‐treated HRCECs was evaluated by colony formation assay, and the number of cell colonies was counted. (F‐G) HRCECs were treated with HG (4.5 mg/mL) without or with different concentrations of arctiin (0.05, 0.5 or 2.0 mg/mL) for 48 h. The cells were immunostained with Ki67 (Red) and DAPI (Blue). Photographs were obtained by confocal microscopy, and the quantification of Ki67‐positive cell was calculated by Zeiss LSCM image examiner software. Scale bar: 50 µm. (H‐I) The formation of capillary‐like structures was photographed with a microscope, and the quantification of total tube length was calculated by ImageJ software. Scale bar: 250 µm. All data are presented as the mean ± SD (*P < .05, ^**^ *P* < .01, ^***^ *P* < .001 vs. HG group)

First, we evaluated the effects of glucose concentration on cell proliferation at different time intervals in human retinal capillary endothelial cells (HRCECs). As shown in Figure 1B, the cell viability of HRCECs induced by different high glucose concentrations was significantly higher than that of HRCECs in low glucose DMEM (NG) at 48 hours and 72 hours, while no significant change was observed among the groups with increased glucose concentrations from 4.5 mg/mL to 9.9 mg/mL at 48 hours and 72 hours. The results were consistent with several other studies that high glucose induced the HRCECs proliferation.^23^, ^24^ In addition, except at 9.9 mg/mL high glucose group enhanced the cell viability of HRCECs moderately, other dose groups did not significantly affect the cell viability of HRCECs at 24 hours. Thus, exposure of HRCECs to 4.5 mg/mL high glucose for 48 hours was used for subsequent experiments. To determine whether arctiin treatment could inhibit high glucose (HG)‐induced cell proliferation, HRCECs were treated with HG in the presence or absence of different concentrations of arctiin. Exposure of HRCECs to arctiin significantly decreased HG‐induced cell proliferation in a dose‐dependent manner (Figure 1C). To further investigate the inhibitory effect of arctiin on HG‐pretreated HRCEC proliferation, a clonogenicity assay and Ki67 (a well‐known cell proliferation marker) immunofluorescence staining assay were performed. Our results showed that compared with HG treatment alone, arctiin treatment significantly reduced colony formation and Ki67‐positive cells (Figure 1D‐G). We next examined whether arctiin affects angiogenesis in HRCECs by performing a tube formation assay. As shown in Figure 1H and I, arctiin abrogated the tube formation induced by HG treatment in HRCECs in a dose‐dependent manner. Together, these findings indicate that arctiin effectively inhibits HG‐induced HRCECs proliferation and angiogenesis in vitro.

### 3.2 Arctiin disrupts cell cycle arrest at the G0/G1 phase in HG‐induced HRCECs via down‐regulation of CDK2, CDK4 and cyclin D1

**FIGURE 2:**
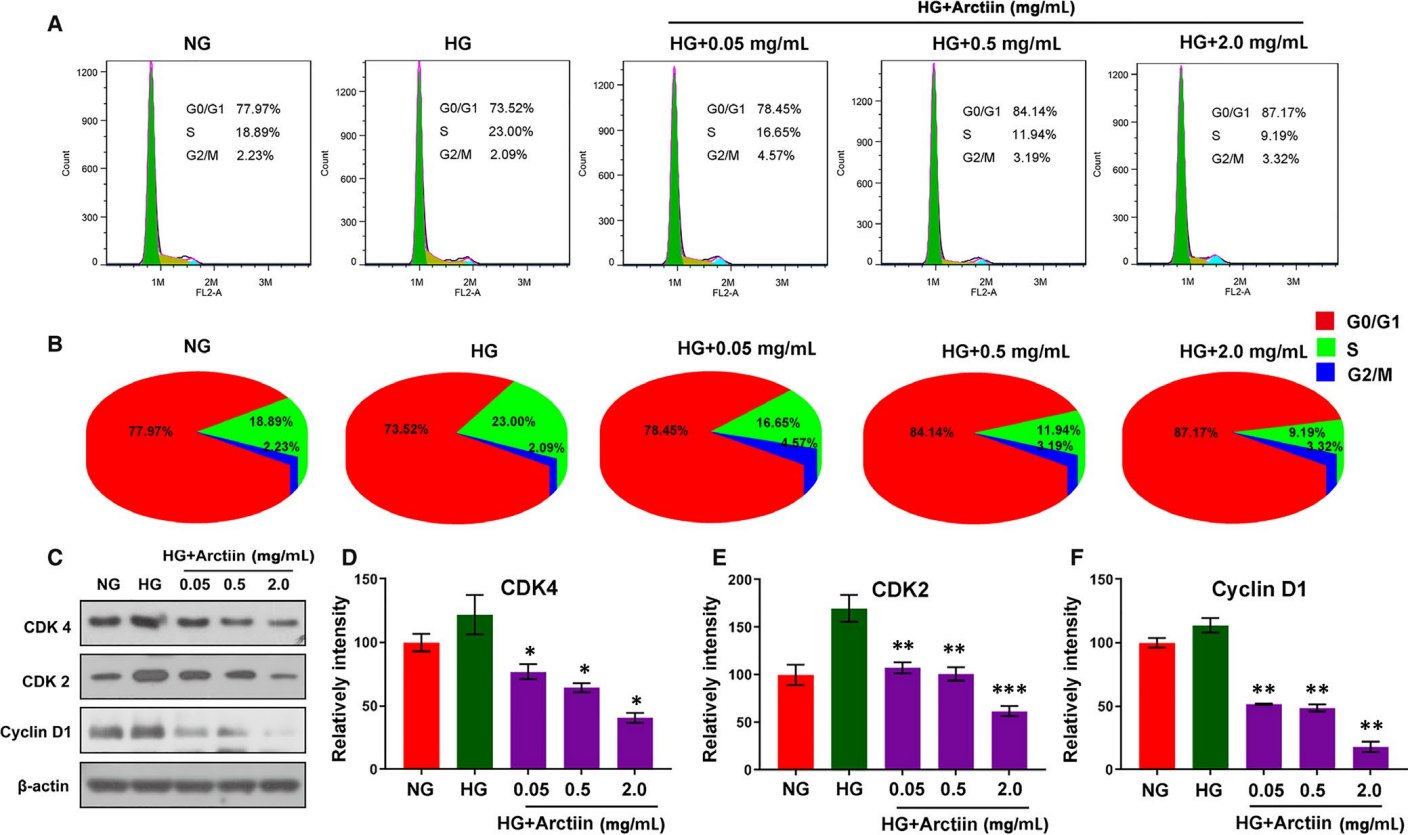
Arctiin disrupts cell cycle arrest at G0/G1 phase in HG‐treated HRCECs. (A‐B) Cells were treated with HG (4.5 mg/mL) and arctiin (0.05, 0.5, or 2.0 mg/mL) for 48 h. The relative ratio of G0/G1, S and G2/M phases was detected using flow cytometry and calculated by FlowJo software. (C‐F) Western blotting analysis of cellular G0/G1 phase regulators CDK4, CDK2 and cyclin D1 under HG and arctiin treatment for 48 h. β‐actin served as the loading control, and the relative quantification of proteins was analysed by Adobe Photoshop software. All data are presented as the mean ± SD (*P < .05, ^**^ *P* < .01, ^***^ *P* < .001 vs. HG group)

Cell cycle arrest is a crucial mechanism involved in the inhibition of cell proliferation.^25^, ^26^ We explored whether the anti‐proliferative effect of arctiin on HG‐induced HRCECs was caused as a result of the cell being arrested at a specific point in the cell cycle. Our flow cytometry results revealed that pretreatment of HRCECs with HG led to increased S phase population and decreased G0/G1 phase population compared with those of the control group. Conversely, arctiin promoted an accumulation of cells at the G0/G1 phase in a dose‐dependent manner (Figure 2A‐B). Further, we carried out Western blot analysis, and the results showed that HG increased the expression of the G0/G1 phase regulators CDK4, CDK2 and cyclin D1 and that these effects were reversed by arctiin treatment (Figure 2C‐F). Together, these findings indicate that arctiin suppressed the transition of HRCECs from the G0/G1 to S phase promoted by HG pretreatment, thereby inhibiting further progress in the cell cycle, and that this cell cycle suppression was accompanied by down‐regulation of the protein levels of CDK4, CDK2 and cyclin D1.

### 3.3 Arctiin attenuates HG‐induced expression of VEGF and perturbation of ROCK1/PTEN/PI3K/Akt signalling pathway in HRCECs

**FIGURE 3:**
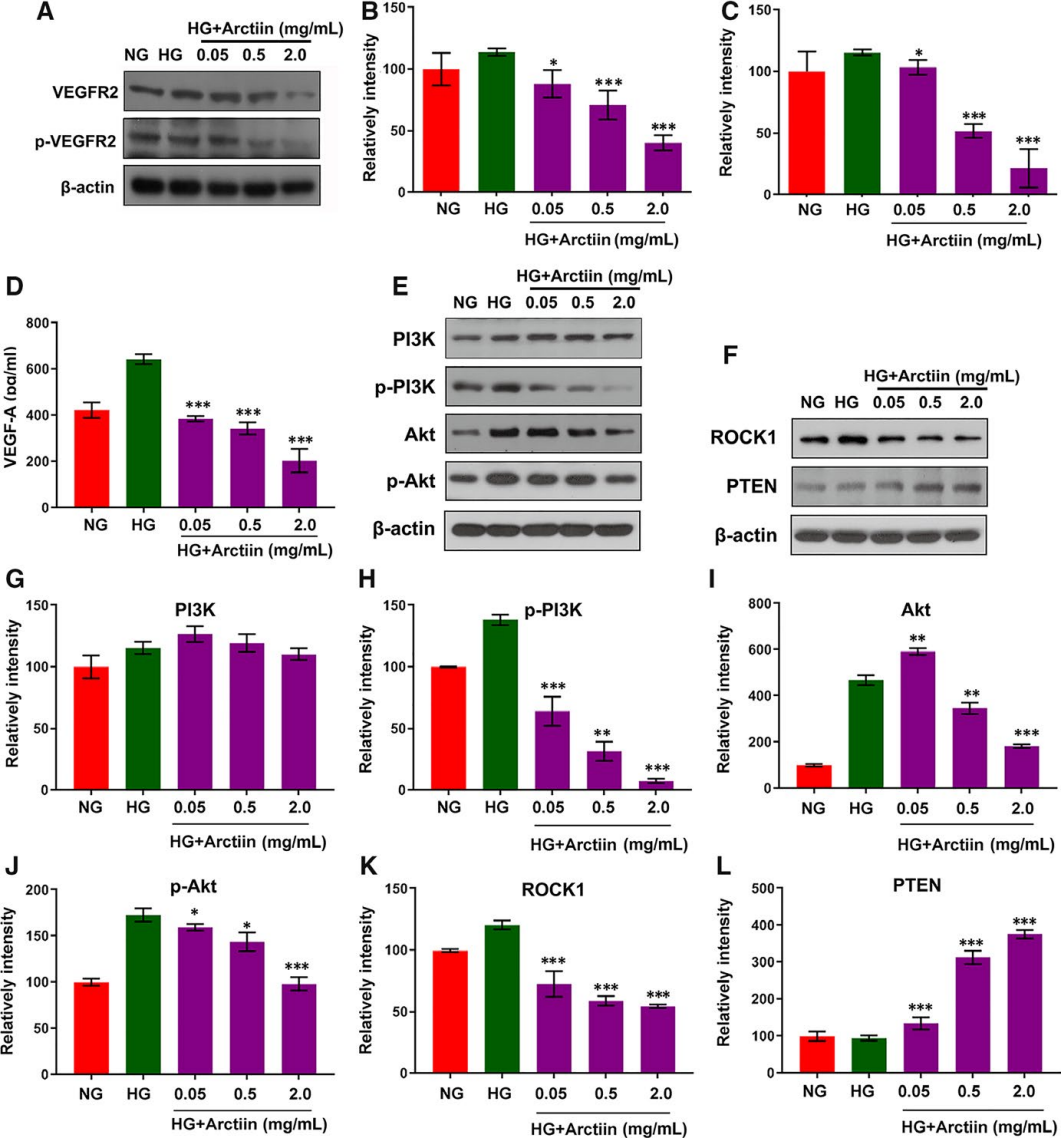
Arctiin attenuates HG‐induced expression of VEGF and perturbation of the ROCK1/PTEN/PI3K/Akt signalling pathway in HRCECs. (A‐C) HRCECs were treated with HG (4.5 mg/mL) in the presence or absence of different concentrations of arctiin and then were characterized by Western blot to assess the expression of VEGFR2 and p‐VEGFR2. β‐actin served as the loading control, and the relative quantification of proteins was analysed by Adobe Photoshop software. (D) The expression level of VEGF‐A by ELISA Kit assay. (E‐L) HRCECs were treated as indicated in (A), and Western blot analysis was used to assess the expression of PI3K, p‐PI3K, Akt, p‐Akt, ROCK1 and PTEN. β‐actin served as the loading control, and the relative quantification of proteins was analysed by Adobe Photoshop software. All data are presented as the mean ± SD (*P < .05, ^**^ *P* < .01, ^***^ *P* < .001 vs. HG group)

The hallmark of DR is the occurrence of retinal neovascularization, and VEGF, the main pro‐angiogenic factor, has been reported to be critically involved in the pathogenesis of DR.^27^, ^28^, ^29^ Therefore, we first examined the effects of arctiin on the expressions of VEGF receptor 2 (VEGFR2) and its phosphorylation form p‐VEGFR2 through Western blot. The results demonstrated that VEGFR2 and p‐VEGFR2 levels were significantly up‐regulated under HG conditions. However, arctiin reversed HG‐induced VEGFR2 and p‐VEGFR2 expression in a dose‐dependent manner (Figure 3A‐C). In addition, we also checked the VEGFA expression level by ELISA kit assay. Consistent with our Western blot results, arctiin also decreased HG‐induced VEGFA expression (Figure 3D).

It has recently been shown that the PI3K/Akt pathway played a major role in angiogenesis, which has been reported to be triggered by growth factors, especially VEGF.^14^ We next investigated the effects of the PI3K/Akt pathway on arctiin‐induced anti‐proliferative activity. Western blot analysis showed that exposure of HRCECs to HG resulted in an apparent increase in the activation/phosphorylation of PI3K and Akt pathways. However, co‐administration of arctiin and HG resulted in the virtual abrogation of the activation/phosphorylation of PI3K and Akt (Figure 3E, G‐J). Studies have shown that PTEN, a tumour suppressor, is a PI3K upstream negative regulator that mediates angiogenesis by activating VEGF expression.^14^ ROCK1 cleavage/activation plays a vital role in the regulation of PTEN activation. The ROCK1 signalling pathway has been implicated in several diseases, such as diabetic nephropathy (DN) and DR, and in the diabetic milieu, high glucose levels can activate the ROCK1 pathway in vascular cells.^30^ We next examined the effects of arctiin on the expression of ROCK1 and PTEN. Treatment with HG led to increased ROCK1 levels and decreased PTEN levels, which were markedly reversed by arctiin in a dose‐independent manner (Figure 3F, K‐L). Such findings suggest that the ROCK1/PTEN/PI3K/Akt/VEGF signalling pathway may be involved in the anti‐proliferative effect of arctiin in HRCECs.

### 3.4 ROCK1 inhibitor Y‐27632 reverses PTEN activation, PI3K/Akt dephosphorylation and VEGF down‐regulation mediated by arctiin

**FIGURE 4:**
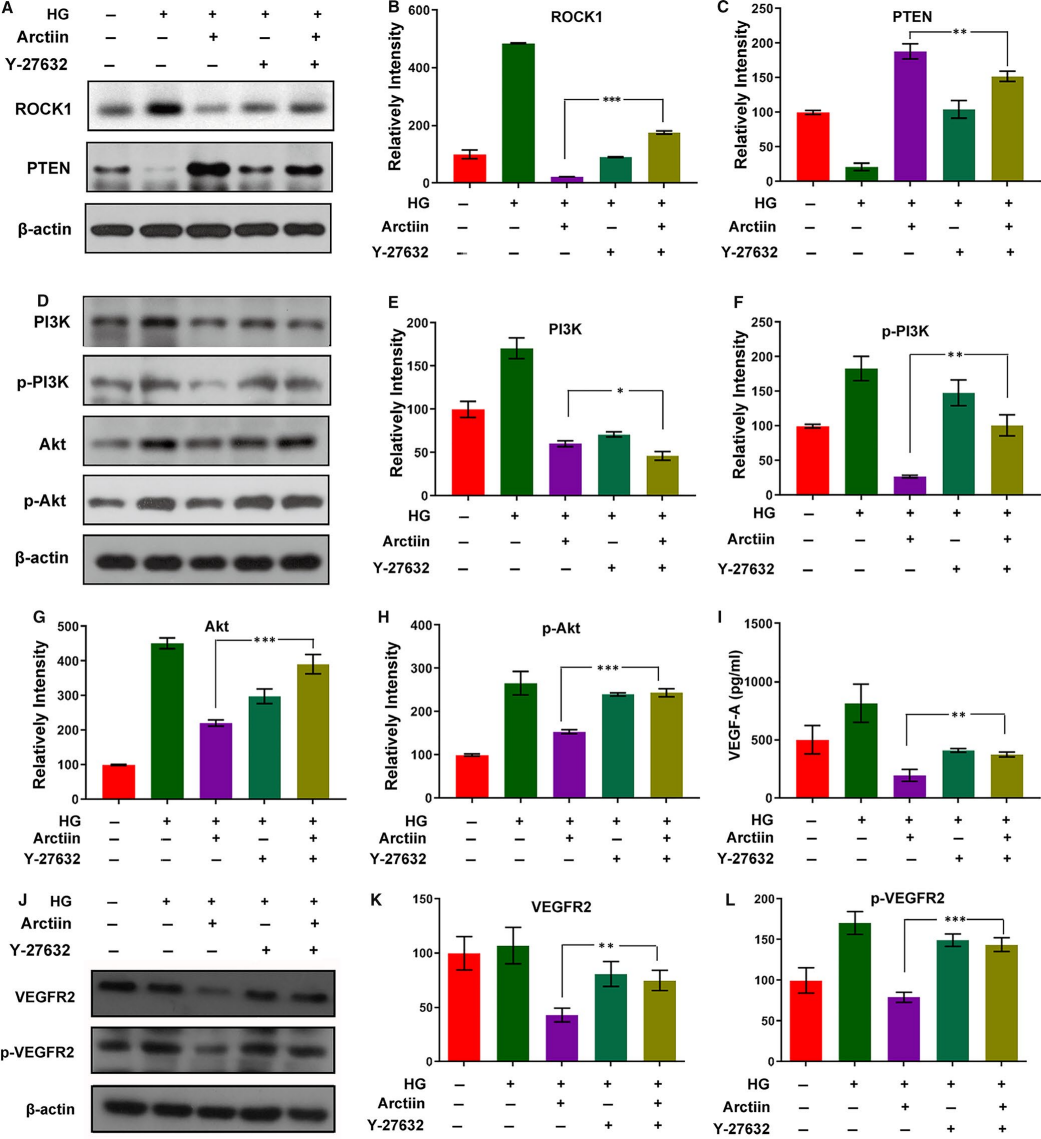
ROCK1 inhibitor Y‐27632 reverses PTEN activation, PI3K/Akt de‐phosphorylation and VEGF down‐regulation mediated by arctiin. (A‐H) HRCECs were pretreated with Y‐27632 (20 μmol/L) for 2 h, followed by treatment with arctiin for 48 h. Western blot was used to assess the expression of ROCK1, PTEN, PI3K, p‐PI3K, Akt and p‐Akt. β‐actin served as the loading control, and the relative quantification of proteins was analysed by Adobe Photoshop software.. (I) HRCECs were pretreated with Y‐27632 (20 μmol/L) for 2 h, followed by treatment with arctiin for 48 h. The cells supernatant were characterized by ELISA Kit assay. (J‐L) HRCECs were treated in (A), and Western blot was used to assess the expression of VEGFR2 and p‐VEGFR2. β‐actin served as the loading control, and the relative quantification of proteins was analysed by Adobe Photoshop software. All data are presented as the mean ± SD (*P < .05, ^**^ *P* < .01, ^***^ *P* < .001)

To investigate whether ROCK1 activation plays a critical role in the regulation of PTEN activity and downstream PI3K/Akt/VEGF signalling during the arctiin‐induced anti‐proliferative effect, a ROCK1 inhibitor, Y‐27632, was employed. In the present study, we employed Western blot analysis and immunofluorescence staining assay to examining the effects of Y‐27632 on the expression of ROCK1, PTEN, phosphor‐PI3K, phosphor‐Akt and VEGF. Western blot analysis indicated that pretreatment of HG‐induced HRCECs with Y‐27632 significantly abrogated arctiin‐induced ROCK1 cleavage/activation (Figure 4A‐C). In addition, pre‐incubation of HG‐induced HRCECs with Y‐27632 partially reversed arctiin‐mediated activation of PTEN, inactivation/dephosphorylation of PI3K/Akt (Figure 4D‐H). The Western blot and ELISA Kit results showed that pre‐incubation of HG‐induced HRCECs with Y‐27632 also partially reversed the down‐regulation of VEGFR2, p‐VEGFR2 and VEGF‐A mediated by arctiin (Figure 4I‐L). Hence, we concluded that arctiin‐attenuated HG‐induced expression of VEGF is dependent on the activation of the ROCK1/PTEN/PI3K/Akt signalling pathway in HRCECs.

### 3.5 ROCK1 inhibitor Y‐27632 abrogates arctiin‐disrupted cell cycle arrest in HG‐induced HRCECs

**FIGURE 5:**
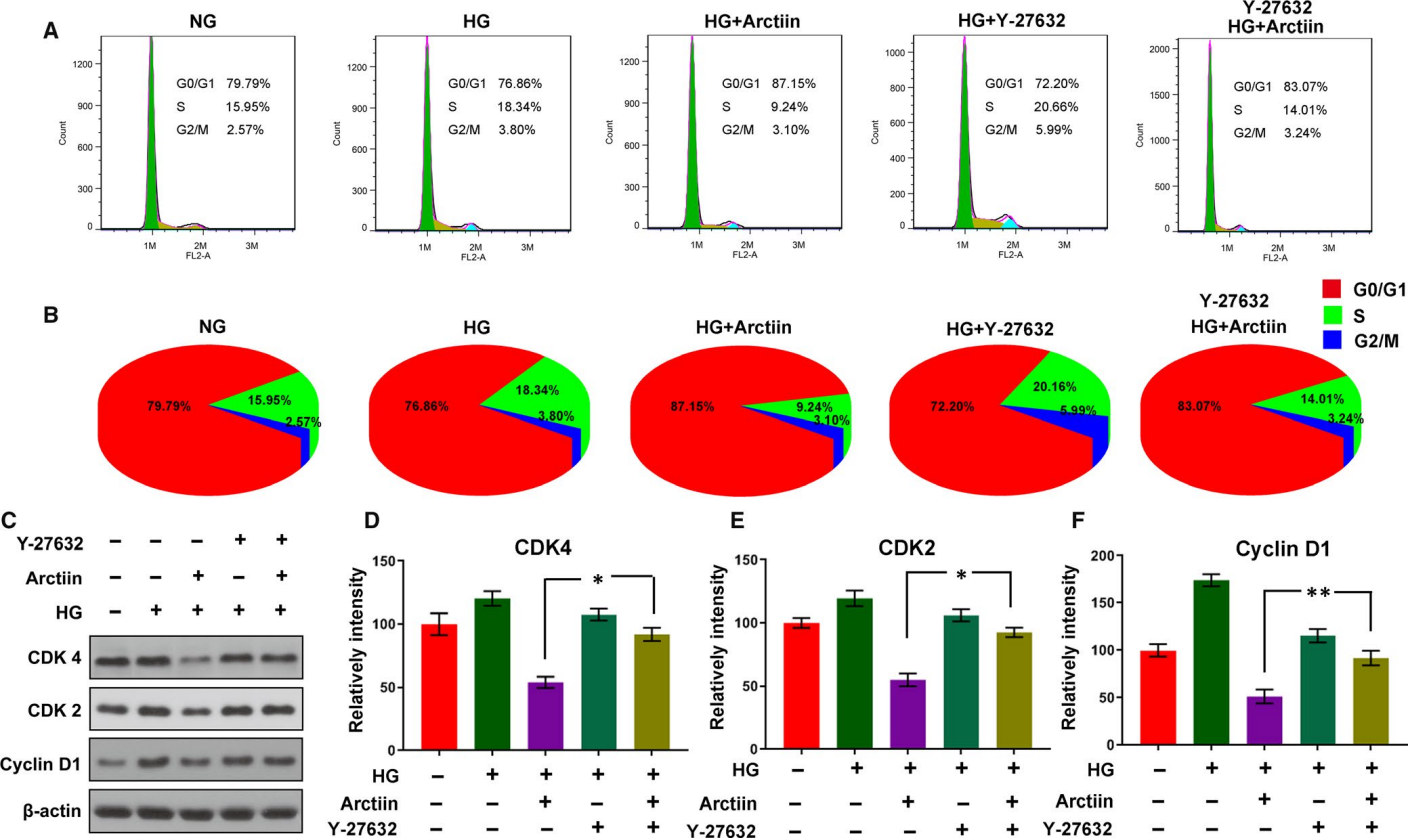
ROCK1 inhibitor Y‐27632 abrogates arctiin‐disrupted cell cycle arrest in HG‐induced HRCECs. (A‐B) HRCECs were pretreated with Y‐27632 (20 µmol/L) for 2 h, followed by treatment with arctiin for 48 h. The cell cycle was analysed by flow cytometry, and the relative ratios of the G0/G1, S and G2/M phases were calculated using FlowJo software. (C‐F) Western blot analysis of cellular G0/G1 phase regulators CDK4, CDK2 and cyclin D1 after pretreatment with Y‐27632 (20 µmol/L) for 2 h, followed by treatment with arctiin for 48 h. β‐actin served as the loading control, and the relative quantification of proteins was analysed by Adobe Photoshop software. All data are presented as the mean ± SD (*P < .05, ^**^ *P* < .01, ^***^ *P* < .001)

We further investigated whether inhibition of ROCK1 activity is responsible for arctiin‐disrupted cell cycle arrest in HG‐induced HRCECs. Our flow cytometry results revealed that pretreatment of HG‐induced HRCECs with Y‐27632 abrogated arctiin‐disrupted cell cycle arrest, as evidenced by an apparent increase in S phase population and a decrease in G0/G1 phase population compared with those in HG‐induced HRCECs treated with arctiin alone (Figure 5A‐B). Moreover, pretreatment of HG‐induced HRCECs with Y‐27632 also attenuated the decrease in levels of CDK4, CDK2 and cyclin D1 mediated by arctiin (Figure 5C‐F). These findings indicate that activation of ROCK1 is required for arctiin‐disrupted cell cycle arrest in HG‐induced HRCECs.

### 3.6 ROCK1 inhibitor Y‐27632 attenuates the anti‐proliferative effect and tube formation inhibiting effect of arctiin in HG‐induced HRCECs

**FIGURE 6:**
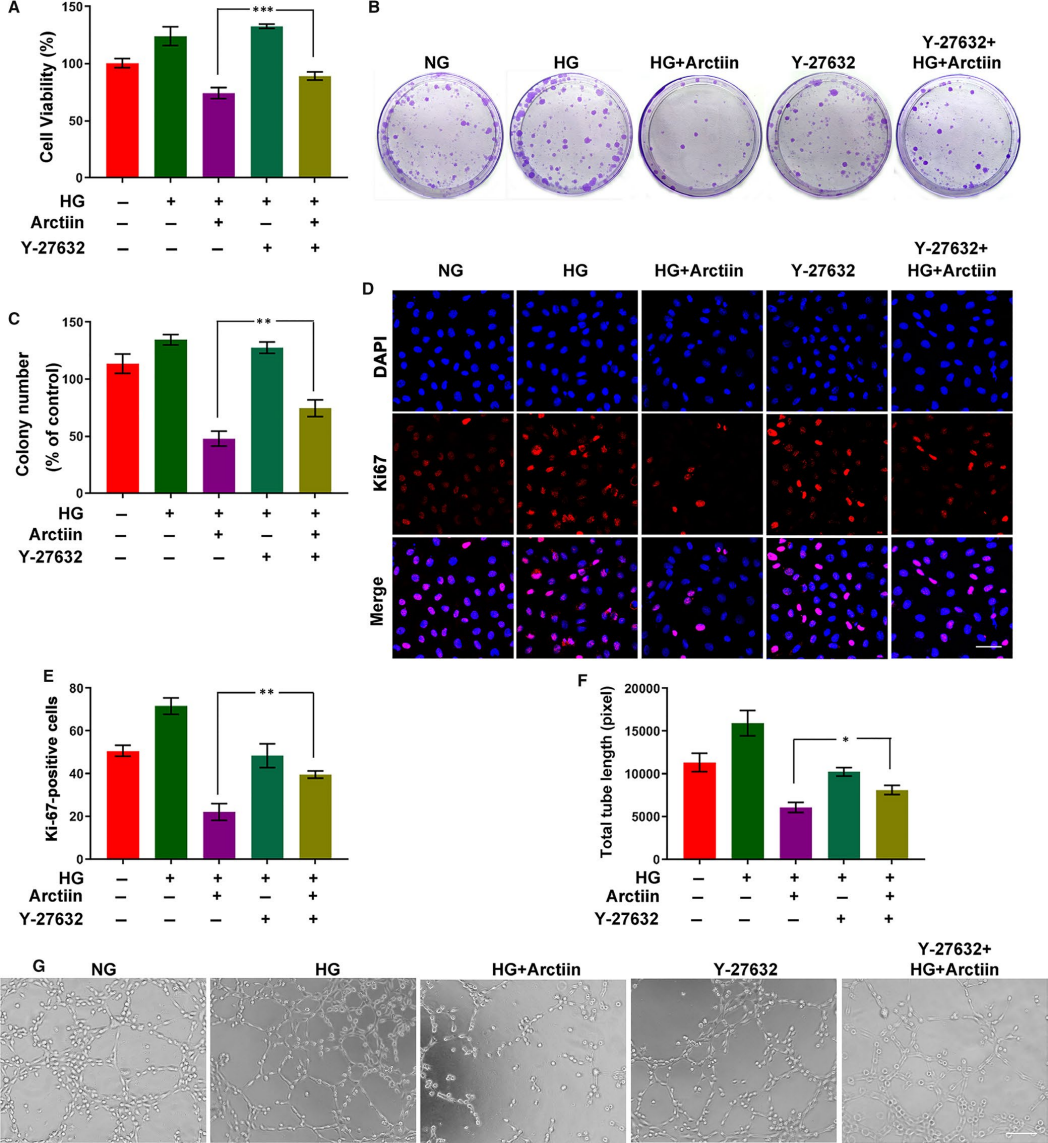
ROCK1 inhibitor Y‐27632 attenuates the anti‐proliferative and tube formation inhibition activity of arctiin in HG‐induced HRCECs. (A) Following pre‐incubation of the cells with Y‐27632 and subsequent treatment with arctiin for 48 h, the effect of arctiin on cell viability in HG‐induced HRCECs was characterized. (B‐C) Following pre‐incubation of the cells with Y‐27632 and subsequent treatment with arctiin for 48 h, cells were cultured for 2‐3 weeks and then stained with crystal violet, the number of cell colonies was counted. (D‐E) After pre‐incubation of HRCECs with Y‐27632, followed by treatment with arctiin for 48 h, HRCECs were immunostained with Ki67 (Red) and DAPI (Blue). Photographs were obtained by confocal microscopy and measured by Zeiss LSM image examiner software. Scale bar: 50 µm. (F‐G) After pre‐incubation of cells with Y‐27632 and subsequent treatment with arctiin for 48 h, the formation of capillary‐like structures was photographed with a microscope, and the quantification of total tube length was calculated by ImageJ software. Scale bar: 250 µm. All data are presented as the mean ± SD (*P < .05, ^**^ *P* < .01, ^***^ *P* < .001)

We next further assessed the role of ROCK1 in arctiin‐inhibited cell proliferation and tube formation. Our MTT assay showed that co‐administration of Y‐27632 and arctiin significantly increased cell viability compared with that of the cells treated with arctiin alone (Figure 6A). The clonogenicity and immunofluorescence staining assay demonstrated that combined treatment with arctiin and Y‐27632 markedly enhanced the colony number and the number of Ki‐67‐positive cells compared with those in the group treated with arctiin alone (Figure 6B‐E). The tube formation assay further confirmed that cotreatment of Y‐27632 and arctiin dramatically reversed arctiin‐mediated tube formation inhibition (Figure 6F‐G). Taken together, these findings demonstrate that ROCK1 activation plays a critical role in arctiin‐inhibited cell proliferation and tube formation in HG‐induced HRCECs.

## 4 DISCUSSION

DR, a type of specific fundus lesion, is one of the most common and severe microvascular complications of diabetes.^31^ DR is mainly due to chronic retinal cell exposure to a high glucose environment, leading to a series of fundus diseases, such as migration and proliferation of endothelial cells, retinal detachment and cell permeability.^32^, ^33^ Among them, neovascularization is the leading characteristic of DR. It has been reported that arctiin, a major lignin component of *Fructus arctii*, has an effect on experimental glomerulonephritis and diabetic nephropathy and can effectively reduce serum glucose levels in diabetic rats.^21^, ^34^ These results suggest that arctiin might be a lead compound for DR therapy. Consistent with these studies, our results indicated that arctiin significantly inhibited the cell proliferation of HRCECs cultured under high glucose conditions. Moreover, arctiin suppresses HG‐induced clonogenicity, reduces the number of Ki67‐positive cells and abrogates tube formation in a dose‐dependent manner. Additionally, arctiin disrupts HG‐induced HRCEC cell cycle arrest at the G0/G1 phase via down‐regulation of the cell cycle regulators CDK4, CDK2 and cyclin D1.

VEGF, a common pro‐angiogenesis factor that regulates vasculogenesis and angiogenesis, is considered one of the most powerful angiogenic factors and has close relations with DR.^35^, ^36^, ^37^ Under normal conditions, the expression of VEGF in the retinal tissue is low, which plays an important role in the formation, stability and maintenance of retinal blood vessels. However, in the hyperglycaemic state, VEGF is up‐regulated and participates in the development of diabetic retinopathy. A previous study indicated that inducing the expression of VEGF promotes the proliferation of endothelial cells and stimulates angiogenesis, which exacerbates DR, and demonstrated that suppression of VEGF signalling inhibits both endothelial cell proliferation and vasculogenesis.^37^ Consistent with previous studies, our finding indicated that the VEGF expression level was up‐regulated under high glucose conditions. As expected, after treatment with arctiin, the expression level of VEGF was down‐regulated in a dose‐dependent manner, which corresponded to the tube formation assay results. Furthermore, it was reported that VEGF exerts pro‐angiogenic activity via binding with high affinity to VEGF receptor 2 (VEGFR2) and inducing the phosphorylation of VEGFR2. In our present studies, we also examined the expression level of VEGFR2 and its phosphorylation form p‐VEGFR2, and their change trends were consistent with VEGF. Such findings suggest that arctiin inhibits HG‐induced HRCEC proliferation through the down‐regulation of VEGF.

Numerous studies reported that the PI3K/Akt signalling pathway is implicated in VEGF expression and in the reduction in angiogenesis in endothelial cells.^38^, ^39^ The up‐regulation of VEGF‐mediated angiogenic effects was dependent on the activation of the PI3K/Akt signalling pathway.^38^ Recent studies indicated that the PI3K/Akt signalling pathway participates in the regulation of endothelial cell proliferation and neovascularization of DR.^40^, ^41^ There is a suggestion that the inhibition of the PI3K/Akt signalling pathway could have beneficial therapeutic effects for the management of DR.^40^ Consistent with these studies, our experiments demonstrated that the PI3K/Akt signalling pathway was activated in HG‐induced HRCECs in vitro, which could be inhibited after treatment with arctiin. These results suggested that arctiin inhibits HG‐induced HRCEC proliferation via down‐regulation of VEGF expression by inactivating the PI3K/Akt signalling pathway.

It has been well documented that PTEN is a negative regulator of the PI3K/Akt pathway,^42^ which plays a critical role in the regulation of angiogenesis in retinal tissues and mediates neovascularization by activating VEGF expression.^37^ ROCK1 (160 kDa) belongs to a family of serine/threonine kinases that are activated via interaction with Rho GTPases or caspase‐3 by cleavage the C‐terminal autoinhibitory domain away (30 kD) from the kinase active site.^7^ Recent evidence revealed that PTEN is a new ROCK1 substrate that is involved in the regulation of cell death and survival.^11^, ^43^ Numerous studies showed that ROCK1 activation enhances the activity of PTEN and inhibits the activation of Akt.^40^ For instance, PTEN phosphorylation induced by ROCK1 decreases the phosphorylation of Akt in HEK cells.^43^, ^44^ In the present study, we found the following evidence showing that ROCK1/PTEN activation and PI3K/Akt inactivation are responsible for arctiin‐inhibited VEGF expression, cell proliferation and tube formation in HG‐induced HRCECs: first, treatment of HG‐induced HRCECs with arctiin results in the activation/cleavage of ROCK1, activation of PTEN and inactivation of PI3K/Akt in a dose‐dependent manner. Second, pretreatment of HG‐induced HRCEC cells with the ROCK1 inhibitor Y‐27632 dramatically abrogated arctiin‐mediated PTEN activation, PI3K/Akt inactivation and VEGF down‐regulation. Third, inhibition of ROCK1 activity by Y‐27632 also attenuated arctiin‐induced cell cycle arrest, cell proliferation inhibition and tube formation inhibition in HG‐induced HRCECs. Taken together, our findings indicate that the ROCK1/PTEN/PI3K/Akt signalling pathway plays a crucial role in arctiin‐inhibited VEGF expression, cell proliferation and tube formation in HG‐induced HRCECs.

## 5 CONCLUSION

**FIGURE 7:**
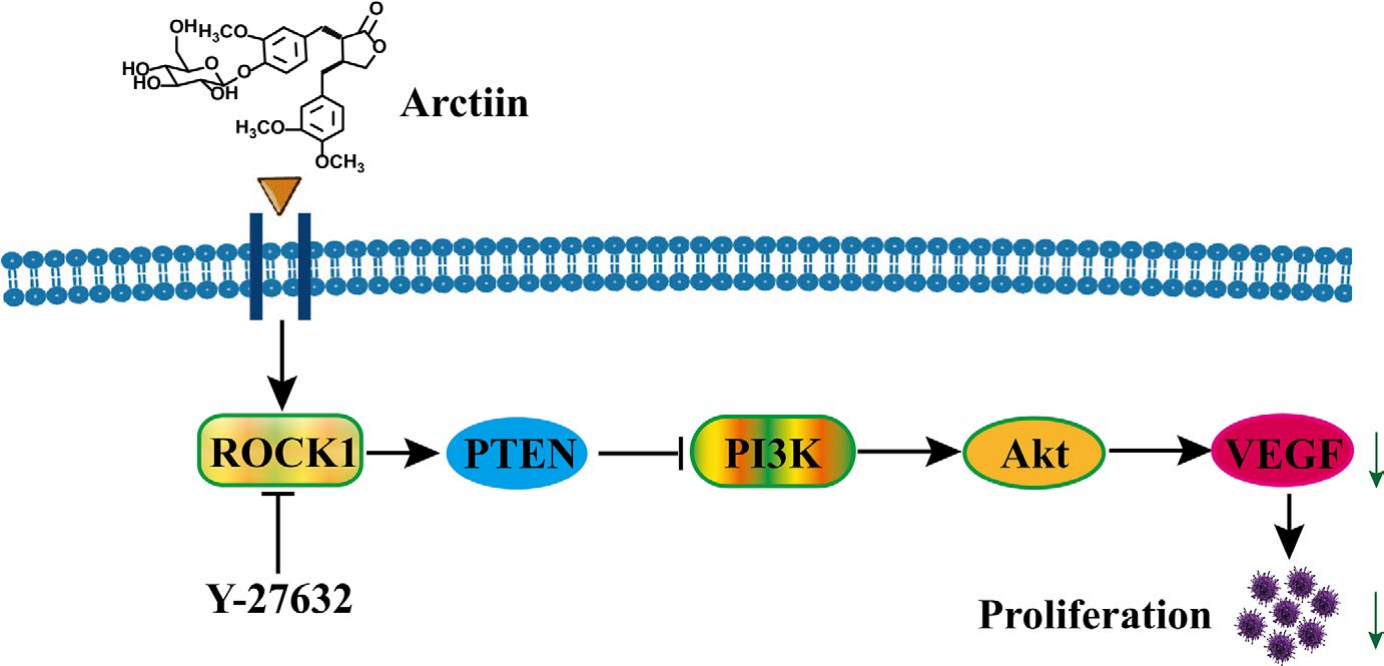
A proposed model for arctiin‐mediated anti‐proliferative effect. Arctiin induced an anti‐proliferative effect in HRCECs, in which ROCK1 activation represents the primary insult, leading in turn to PTEN activation and PI3K inactivation and then to Akt inactivation, which results in down‐regulation of VEGF and ultimately inhibition of HRCEC proliferation

In summary, the present studies indicated that arctiin effectively inhibits high glucose‐induced human retinal capillary endothelial cell proliferation via ROCK1/PTEN/PI3K/Akt/VEGF signalling pathway (Figure 7). Collectively, these findings suggest a hypothetical model of arctiin‐induced anti‐proliferative effects in HG‐induced HRCECs. In this model, arctiin induces ROCK1/PTEN activation, leading to PI3K/Akt inactivation, VEGF down‐regulation and ultimately the inhibition of HG‐induced HRCEC proliferation. Further efforts to understand the mechanisms by which arctiin induces an anti‐proliferative effect in HG‐induced HRCEC both in vitro and in vivo could improve preventive treatment outcomes for DR and potentially for other diabetic complications as well.

## CONFLICT OF INTEREST

The authors confirm that there are no conflicts of interest.

## AUTHOR CONTRIBUTIONS

LCL and GBL contributed to the study design; MZ and HYZ contributed data; MZ and LCZ drafted the statistical analysis plan; MZ and GBL wrote the manuscript.

## Data Availability

The data that support the findings of this study are available from the corresponding author upon reasonable request.
